# NEWS

**DOI:** 10.7189/jogh.02.010203

**Published:** 2012-06

## ENVIRONMENT

• The 18 past winners of the Blue Planet prize – which is the unofficial Nobel prize for the environment – warned in February that civilization is faced with a “perfect storm” of ecological and social problems, driven by overpopulation, overconsumption and environmentally malign technologies. In the face of an “absolutely unprecedented emergency”, they said that society had “no choice but to take dramatic action to avert a collapse of civilization”. (*The Guardian*, 20 Feb 2012)

• A traditional root crop long neglected by modern science could be the best bet for farmers in Sub-Saharan Africa to beat climate change, according to a new study published in a special edition of Tropical Plant Biology. This study found that the rugged root crop cassava could brush off expected temperature rises of up to 2°C in the region by 2030 – and could be even more productive thanks to climate change. (*AllAfrica*, 28 Feb 2012)

• Speaking in Bangkok at the Southeast Asia launch of the IPCC special report on managing the risks of extreme events and disasters, Rajendra K. Pachauri, chief of the Intergovernmental Panel on Climate Change (IPCC), said that “…The world can no longer ignore the facts about climate change and its link to human society”. He added that “…some facts which are incontrovertible need to be accepted by the public”. Pachauri said he believed the upcoming Rio +20 sustainability conference in June 2012 would lead to new agreements that could help shift the world onto a path of more sustainable development. (*AlertNet*, 04 May 2012)

• According to a new report from the United Nations Environment Programme, tens of millions of new jobs could be created around the world in the next two decades if green policies are put in place to switch the high-carbon economy to low-carbon. The report estimated that between 15 and 60 million additional jobs would likely be created as net gains in employment for the world economy, taking into account any job losses in high-carbon industries. As well as generating net new gains in the number of jobs, the switch to a green economy could help to lift millions of people out of poverty. (*The Guardian*, 31 May 2012)

• The UN’s Environment Programme sounded the alarm in its fifth Global Environment Outlook (GEO-5) report, published two weeks before the Rio +20 summit in Brazil, one of the biggest environmental meetings in years. Population growth, urbanization and consumption are set to inflict irreversible damage on the planet, and the UN called for urgent agreement on new environmental targets at an Earth summit in June 2012. Only a few hours after GEO-5′s release, the journal *Nature* published a review of scientific evidence on environmental change concluding that the biosphere – the part of the planet that supports life – “…could be heading for rapid, possibly irreversible change”. (*Reuters*, 06 Jun 2012)

## DEMOGRAPHY

• India is the most dangerous place in the world to be a baby girl. Newly released data shows that an Indian girl child aged 1-5 years is 75% more likely to die than an Indian boy, making this the worst gender differential in child mortality for any country in the world. (*Times of India*, 01 Feb 2012)

• The call to invest in adolescent girls has been voiced within the development field in recent years, supported by the UK Department for International Development (DfID), the World Bank and several UN agencies. “Girl effect” proponents argue that if girls in developing countries delay childbearing they will be significantly better off. However, this argument is based on questionable evidence, because the causal role of early childbearing in poverty has not been convincingly demonstrated. (*The Guardian*, 10 Feb 2012)

• A major report from the Royal Society suggested that the World population needs to be stabilised quickly, and high consumption in rich countries rapidly reduced, to avoid “…a downward spiral of economic and environmental ills”. Their assessment of humanity's prospects in the next 100 years, which has taken nearly 2 years to complete, argued strongly that to achieve long and healthy lives for all 9 billion people expected to be living in 2050, the entangled issues of population and consumption must be pushed to the top of political and economic agendas.

• Last year, the world population reached 7 billion, adding the last billion in merely 12 years. Despite this rapid growth, the predictions about the potentially disastrous consequences of rapid population growth have not materialized; in fact, various summary measures of individual well-being have in fact increased. From 1960 to 2010, global life expectancy increased from 51.2 to 67.9 years, infant and maternal death rates declined substantially, education and levels of female schooling increased, global per capita food production and consumption rose, and the proportion of the global population living in poverty declined significantly. (*Slate*, 03 May 2012)

• According to the United Nations, about 3.4 billion people live in urban areas – about half of the world's population – but nearly a third of them, or about one billion, live in slum conditions. By 2030 that number is likely to double, unless living conditions improve. Concern has been mounting so much about the situation that the *Lancet* has set up a Commission on Healthy Cities to look at what should be done. (*BBC News*, 30 May 2012)

## ECONOMY

• After a decade of rapid economic growth, many developing countries have attained middle-income status based on increase in their overall GDP, but poverty reduction has not kept pace with their GDP growth. As a result, most of the world’s poor – up to a billion people – now live in these new middle-income countries (MICs), making up a “new bottom billion”, shifting the majority of the global disease burden into MICs. This poses a challenge to global health agencies, which are accustomed to disbursing funds on the assumption that the majority of poor people live in poor countries. (*Center for Global Development*, 10 Jan 2012)

• Ratings agency Moody's maintained France’s top AAA credit rating for now, but the country was downgraded by another agency, Standard & Poor's (S&P). Moody's said it would update its position on France later this quarter. It is feared that downgrading of France's credit rating would further increase debt worries across Europe. (*BBC*, 16 Jan 2012)

• The World Bank (WB) at the end of February 2012 that the share of people living in extreme poverty around the world continued to decline in recent years, despite financial crises and surging food prices. The WB’s preliminary estimates for 2010 showed that the world’s extreme poverty rate – people living below US$ 1.25 a day – had fallen to less than half of its 1990 value, meeting the first UN’s Millennium Development Goal of halving extreme poverty from its 1990 level before its 2015 deadline. In 2008, about 1.29 billion people – roughly 22% of the developing world’s population – had less than US$ 1.25 a day to make their living, whereas 17 years earlier 1.94 billion people lived in extreme poverty. These estimates are based on more than 850 household surveys in about 130 countries. The region with the highest extreme poverty rate was Sub-Saharan Africa, where about 47% of the population had below US$ 1.25 a day to live. (*Wall Street Journal*, 29 Feb 2012)

• Recently, 65 world-renowned researchers, economists and Nobel laureates got together to answer what would they do if they had US$ 75 billion and four years to improve the world's well-being. They released their findings in April this year, after more than a year of reviewing proposals and evidence, thanks to the Copenhagen Consensus Center. Being economists, they weighed their choices carefully using cost-benefit analyses. Seventy-five billion dollars represents a 15% annual increase on top of the current investments of developed nations in foreign aid. They agreed that child nutrition is the “best buy” in development today.

• The idea that an infusion of hope can make a big difference to the lives of poor people was the central idea of a lecture at Harvard University by Esther Duflo, an economist at the Massachusetts Institute of Technology known for her data-driven analysis of poverty. Ms Duflo argued that the effects of some anti-poverty programmes go beyond the direct impact of the resources they provide. They make it possible for the very poor to hope for more than mere survival. (*The Economist*, 12 May 2012)

## ENERGY

• The International Year of Sustainable Energy for All kicked off in January 2012. United Nations officials called on governments, the private sector and civil society to help expand access to energy, improve efficiency and increase the use of renewables. Globally, one person in five still lacks access to modern electricity, while three billion people still use wood, coal, charcoal, or animal waste for cooking and heating. UN Chief Mr. Ban attended the opening of the World Future Energy Summit, which is taking place in Abu Dhabi, United Arab Emirates. (*UN News*, 16 Jan 2012)

• A new UN report suggests that women should be the focus of efforts to bring access to modern energy to those who lack it, as “...bringing energy to women and girls helps lift communities out of poverty and improves health”. But the report also warned that providing energy alone was not enough to combat poverty. Programmes to provide energy access work best when they are paired with access to other key services, such as education and microfinancing. (*The Guardian*, 19 Jan 2012)

• Royal Dutch Shell and other natural resources companies have stepped up efforts to counteract planned anti-corruption rules that would force them to disclose payments to governments in countries where they operate. Bill Gates recently threw his weight behind a proposed rule included in the Dodd-Frank act, which would require US extractive companies to disclose similar payments. (*Financial Times*, 19 Feb 2012)

• US-based *d.light* design company was one of the pioneers in distributing rugged solar lamps and lanterns, and now distributes its products in 40 countries, focusing particularly on Sub-Saharan Africa and India. In just five years, the company has distributed more than 1.4 million lanterns, ranging in price from about US$ 10 for a student lamp to about US$ 45 for a rugged, handheld lantern with four light settings and cell-phone charger. A partnership with the Shell Foundation is aimed at implementing market awareness programs and supporting local entrepreneurship. Donn Tice, chief executive officer, said that “…while *d.light* is a for-profit company, it has a social mission to help people replace kerosene lanterns, cheap flashlights and other throwaway items with safer, cleaner, more permanent lanterns.” (*National Geographic News*, 06 Jun 2012)

• The poll by the AP-NORC Center for Public Affairs Research showed that, when it comes to saving energy, people in the United States know that driving a fuel-efficient car accomplishes more than turning off the lights at home, but that doesn't mean they'll do it. A new poll shows that while most of those questioned understand effective ways to save energy, they have a hard time adopting them. Six in 10 surveyed say driving a more fuel-efficient car would save a large amount of energy, but only 1 in 4 says that's easy to do. People also are sceptical of carpooling or installing better home insulation, rating them as effective but impractical. On the other end of spectrum, 8 in 10 say they easily can turn off the lights when they leave a room, and 6 in 10 have no problem turning up the thermostat in summer or down in winter, although fewer than half think those easy steps save large amounts of energy. (*KnoxNews*, 09 Jun 2012)

## PEACE & HUMAN RIGHTS

• About 200 million people around the world use illicit drugs. Cannabis users comprise between 125–203 million, users of opioids (heroin and morphine), amphetamines or cocaine total 15–39 million; and those who inject drugs numbered between 11–21 million. Ecstasy, LSD, non-medical use of prescription drugs and anabolic steroids are not included in this estimate. (*The Lancet*, 06 Jan 2012)

• A rising proportion of abortions worldwide are putting women's health at risk. The World Health Organization study estimates that global abortion rates are steady, at 28 per 1000 women each year. *The Lancet*, which published the study, characterized the figures as “deeply disturbing”. (*BBC News*, 19 Jan 2012)

• UN human rights experts have expressed their dismay at what they see as the continuing abuse of anti-terrorism legislation to curb freedom of expression in Ethiopia. The blunt criticism from the UN comes after a Human Rights Watch also accused the government of forcibly relocating thousands of people in the Gambella region. (*The Guardian*, 03 Feb 2012)

• The global arms trade has grown by nearly a quarter over the last four years, with new growth mainly in poorer countries. India is now officially the world's biggest importer of arms. (*The Guardian*, 19 Mar 2012).

• The UN refugee agency predicted that the number of people fleeing their homes and becoming refugees or displaced in their own countries will increase in the next 10 years. This will come as a result of a multitude of complex causes, ranging from conflict and climate change to population growth and food shortages, according to their report. (*Associated Press*, 01 Jun 2012)

## FOOD, WATER AND SANITATION

• Jose Graziano da Silva of Brazil, the new FAO Chief from the start of 2012, said that volatility in food markets was likely to continue and that prices “will not be going up as in the last two to three years but will also not drop down” (*Reuters*, 03 Jan 2012)

• The United Nations World Food Programme (WFP) is launching a week-long campaign in early February, during which users of the popular online trivia game ‘Freerice’ can recruit their friends to help bring food to the world's most vulnerable populations. Under its theme ‘6 Degrees of Freerice’, fans of the game are being asked to recruit six friends to join in the online fight against hunger. (*UN News*, 01 Feb 2012)

• Better access to water and sanitation is crucial to reducing maternal mortality and achieving Millennium Development Goal 5, according to a group of scientists from McMaster University in Canada. The impact of unsafe water and sanitation on the death rates of children under the age of five and mothers in the year after childbirth have been quantified for the first time. (*Environmental Health*, 17 Feb 2012).

• It has been estimated that alcohol kills more than 2.5 million people annually, more than AIDS, malaria or tuberculosis – making it one of the world’s leading killers. For middle-income people, who constitute half the world's population, alcohol is the top health risk factor, greater than obesity, inactivity and even tobacco. (*Scientific American*, 15 Feb 2012)

• The UN announced that international target to halve the number of people who do not have access to safe drinking water has been met five years before the 2015 deadline. According to the WHO and UNICEF’s joint monitoring programme for water supply and sanitation (JMP), between 1990 and 2010 more than 2 billion people gained access to improved drinking water sources, such as piped supplies and protected wells. Using data from household surveys and censuses, 89% of the population – 6.1 billion people – now used improved drinking water sources at the end of 2010, 1% more than the 88% target contained in Millennium Development Goal 7 (MDG7), set in 2000. (*The Guardian*, 06 Mar 2012)

## SCIENCE & TECHNOLOGY

• Tiny capsules engineered to mimic part of the body's immune system could strengthen its response to vaccines. The nanoparticles, described in the journal Nature Materials, are a message sent from cells in the skin to warn of a threat. Scientists from Duke University in the USA said mice given them as part of a vaccine coped with otherwise lethal infections. They could soon be suitable for humans, too. Vaccination involves priming the immune system to recognise particular bacteria or viruses, so that it is ready to counter-attack quickly in the event of a genuine infection. (*BBC News*, 22 Jan 2012)

• A two-year study of nearly 200 000 American girls and women aged 9 to 26 showed that those who received the HPV vaccine Gardasil were not at a greater risk for 16 different autoimmune disorders. (*PressTV*, 29 Jan 2012)

• The full details of recent experiments that made a deadly flu virus more contagious will be published, despite recommendations by the United States that some information be kept secret for fear that terrorists could use it. The WHO announcement made in February followed two months of heated debate about whether the results of the research should be published. Anthony S. Fauci, director of the US National Institute of Allergy and Infectious Diseases, said that “…the group consensus was that it was much more important to get this information to scientists in an easy way to allow them to work on the problem for the good of public health”. WHO spokesman Gregory Hartl told Reuters that “…there must be a much fuller discussion of risk and benefits of research in this area and risks of virus itself”. Critics said this was a closed meeting, dominated by flu people who have a vested interest in continuing this kind of work. (*New York Times*, 18 Feb 2012)

• The number of patents filed by large pharmaceutical companies has dropped significantly in recent years. This suggests intensifying problems for the industry to maintain the pipeline of new products over the coming decade. There is also a shift from ‘small molecules’, or chemical-based medicines, to those that are biological and which comprised 60% of the total by 2009. (*Financial Times*, 18 Mar 2012)

• Researchers at Texas A & M University have genetically engineered a goat so it creates malaria vaccine in its milk. Their research is still in a preliminary stage and requires the analyses of safety and effectiveness. Their idea would be to place vaccine producing goats in villages around Africa, so that people could simply drink goats' milk and get immunized. (*Care2*, 16 Mar 2012)

